# Elevating image segmentation with multilevel two-dimensional quantum representation

**DOI:** 10.1371/journal.pone.0331912

**Published:** 2025-09-18

**Authors:** Adel A. Bahaddad, Sayed Abdel-Khalek, Salem Alkhalaf, Hanadi M. AbdelSalam, Anis Ben Ishak, Mersaid Aripov

**Affiliations:** 1 Faculty of Computing and IT, King Abdul-Aziz University, Jeddah, Saudi Arabia; 2 Department of Mathematics and Statistics, College of Science, Taif University, Taif, Saudi Arabia; 3 Department of Information Technology, College of Computer, Qassim University, Buraydah, Saudi Arabia; 4 College of Sciences and Human Studies, Prince Mohammad Bin Fahd University, Al-Khobar, Saudi Arabia; 5 Department of Quantitative Methods, Higher Institute of Management, University of Tunis, Tunis, Tunisia; 6 University of Manouba, ESCT, QuAnLab LR24ES21, Campus Universitaire, Manouba, Tunisia; 7 Department of Applied Mathematics and Computer Analysis, Faculty of Mathematics, NUU, Tashkent, Uzbekistan; Ningbo University, CHINA

## Abstract

In the rapidly advancing field of image analysis and processing, accurately segmenting images into meaningful regions remains a critical challenge. Drawing from recent advancements in quantum computing and information theory, our research introduces an innovative approach to image segmentation. This work presents a novel multilevel segmentation method that utilizes a two-dimensional quantum image representation, offering a more sophisticated and efficient technique for image thresholding. In this framework, the image’s 2D histogram is treated as a quantum system, with quantum Rényi entropy used to quantify the information contained within the image. To enhance segmentation quality, we first improve the contrast of the images by applying a new contrast enhancement algorithm before performing the segmentation. The resulting entropy-based fitness function is then optimized using Differential Evolution (DE) and Particle Swarm Optimization (PSO) algorithms to determine the optimal thresholding values. A comprehensive comparative analysis is conducted between the proposed quantum method and traditional classical approaches, evaluated on a set of benchmark images using nine metrics, including the Wilcoxon test for statistical significance. Experimental results demonstrate the effectiveness of the PSO optimizer, the superiority of the two-dimensional quantum image representation.

## 1 Introduction

Over the past decades, digital image processing has evolved dramatically, driven by its vital role in domains such as medical diagnostics, remote sensing, and autonomous systems [1]. This broad discipline covers tasks like restoration, enhancement, compression, and analysis. Among these, image analysis—particularly edge detection, texture classification, and thresholding-based segmentation—is critical for extracting meaningful information from visual data [2–4].

Image segmentation, in particular, plays a fundamental role by partitioning an image into non-overlapping regions according to homogeneity criteria [5]. Techniques like region growing, clustering, and thresholding are widely used for this purpose [6]. Among them, thresholding is particularly attractive due to its conceptual simplicity and computational efficiency. Traditional approaches, such as Otsu’s method [7] and Kapur’s entropy-based method [8], rely on one-dimensional (1D) histograms to compute optimal thresholds based on between-class variance or Shannon entropy. These methods can be extended from bilevel to multilevel segmentation, which is especially useful for analyzing complex or textured images [9].

However, a major limitation of 1D histogram-based techniques is their inability to incorporate spatial information, which can be crucial for distinguishing between regions with similar intensity distributions but different contextual patterns [10]. To overcome this, Abutaleb introduced 2D histogram-based thresholding in 1989, integrating gray-level intensity and local neighborhood averages [11]. Since then, numerous studies have demonstrated the robustness of 2D histogram-based methods, especially in scenarios involving noise and low contrast [12–16].

Despite their improved segmentation quality, 2D histogram methods introduce a significant computational burden, particularly for multilevel thresholding. As the number of thresholds increases, the search space grows exponentially, rendering exhaustive search impractical. This challenge has led to the adoption of metaheuristic optimization algorithms, such as Particle Swarm Optimization (PSO), Differential Evolution (DE), Genetic Algorithms (GA), and Cuckoo Search (CS), to efficiently navigate high-dimensional search spaces [17,18]. PSO, for instance, offers a good balance between exploration and exploitation, while DE is valued for its robustness and convergence speed in noisy and constrained environments [19–21].

Recent advancements have further enhanced these algorithms through adaptive parameter control, hybrid models with machine learning components, and the integration of quantum-inspired mechanisms [22–25]. However, classical metaheuristics are still constrained by the exponential nature of the search space in multilevel segmentation and may suffer from premature convergence or suboptimal local minima in complex landscapes.

To address these limitations, this study explores a novel segmentation framework that bridges classical and quantum paradigms. We extend the concept of 2D histograms to the quantum domain and employ quantum Rényi entropy as a fitness function. By leveraging metaheuristics such as PSO and DE within this quantum-inspired framework, our approach aims to achieve more precise and computationally efficient multilevel thresholding. This hybrid methodology opens new avenues for enhancing segmentation performance, particularly in challenging scenarios involving noise, low contrast, or intricate textures.

The remainder of this paper is organized as follows: Sect 2 presents the related work, discusses their limitations, and highlights the contributions of this study. Sect 3 details the proposed method and the tools employed. Sect 4 provides an analysis of the experimental results from the comparative study, and Sect 5 concludes the paper by summarizing key findings and outlining future research directions.

## 2 Related work and contribution

This section reviews the evolution of multilevel thresholding methods, with a focus on 2D histogram-based segmentation and its integration with metaheuristic algorithms. We also examine recent advances in quantum image processing and clarify the specific limitations in existing methods that our work addresses. Finally, we present our novel contribution, which builds a bridge between classical thresholding and quantum-enhanced optimization.

### 2.1 Classical 2D thresholding and metaheuristics

This study builds on the foundational work of Abutaleb [11], who first introduced 2D histogram-based thresholding by incorporating local spatial context into gray-level distributions. His approach greatly improved segmentation quality compared to 1D histograms, particularly in noisy or low-contrast environments. Sarkar et al. [26] later extended this technique to multilevel thresholding, enabling more granular partitioning of image content for applications in complex domains such as medical imaging.

More recently, Ben Ishak [27] proposed a refined 2D multilevel thresholding method using Tsallis and Rényi entropy, yielding more adaptive and informative segmentations. His work also explored the integration of quantum genetic algorithms into this framework, showing the potential of hybrid approaches to improve threshold selection. In parallel, Zhang et al. [28] and Sahoo et al. [29] developed alternative 2D histogram construction techniques that enhanced the representational power of pixel distributions, laying the groundwork for more accurate and efficient segmentation.

To cope with the increasing computational complexity of multilevel segmentation, especially with high-resolution images, numerous studies have employed metaheuristic algorithms such as Particle Swarm Optimization (PSO), Differential Evolution (DE), and Genetic Algorithms. These methods help avoid the combinatorial explosion inherent in exhaustive search. Nevertheless, they come with trade-offs such as sensitivity to parameter settings and risks of premature convergence.

### 2.2 Emergence of quantum image processing

Quantum computing opens a promising new frontier for image representation and processing. By encoding classical pixel values into quantum states, quantum image representations can achieve superior parallelism and data compression. Fei et al. [30] reviewed several quantum representations and proposed various quantum-inspired processing tasks, while the use of Von Neumann entropy in image thresholding [31] highlighted the potential of quantum entropy measures for segmentation.

One of the most widely adopted frameworks is the Flexible Representation of Quantum Images (FRQI), introduced by Le et al. [32]. FRQI allows both gray-level intensities and spatial information to be encoded efficiently into quantum states. This model has proven useful in fields such as medical imaging, where data volume and precision are critical. Building on this, Du et al. [33] and Ben Ishak [27] showed that quantum frameworks, when combined with entropic measures and heuristic search, could outperform classical methods in specific contexts.

### 2.3 Limitations of existing methods

Despite these advancements, several limitations persist. Classical 2D thresholding methods are computationally intensive as the number of thresholds increases, making them less suitable for real-time or high-resolution applications. Metaheuristic algorithms, while powerful, often suffer from premature convergence or require extensive parameter tuning, which limits their generalizability and scalability.

On the other hand, most existing quantum thresholding approaches are limited to 1D histogram frameworks or operate in simplified settings. They often neglect richer spatial context and lack integration with advanced optimization strategies. Moreover, quantum entropy metrics like Rényi entropy remain underexplored in this domain, especially when used in conjunction with quantum image representations such as FRQI.

### 2.4 Motivation and contribution

This work aims to bridge the gap between classical and quantum paradigms in image segmentation by proposing a quantum-enhanced multilevel thresholding method based on the FRQI representation. Specifically, we:

- Introduce a novel use of FRQI to encode 2D histograms of classical images as quantum states.
- Apply quantum Rényi entropy as a thresholding criterion within this quantum framework.
- Integrate two well-established metaheuristic algorithms, DE and PSO, to optimize threshold selection in the quantum domain.

Our approach builds on the framework of Al-Mansor et al. [34], who demonstrated how FRQI can encode spatial and intensity information efficiently. We extend their work by incorporating Rényi entropy and heuristic optimization for multilevel thresholding. Furthermore, we complement recent studies like that of Tariq Jamal et al. [35], who compared classical and quantum segmentation techniques using 1D histograms, by advancing to 2D representations and entropy-based quantum optimization.

By systematically comparing classical and quantum formulations, and evaluating the performance of PSO and DE in both contexts, this study sheds light on the concrete benefits of quantum image representations for multilevel thresholding. Our results highlight how such representations, when combined with robust metaheuristic optimization and Rényi entropy, lead to significant improvements in segmentation accuracy, stability across runs, and computational efficiency, thus demonstrating the practical advantages of quantum-inspired approaches over classical techniques.

## 3 The proposed method and used tools

In this section, we explore the segmentation approach in detail. We begin by introducing the concept of quantum image representation within a two-dimensional framework. Next, we present the formulation of classical and quantum Rényi entropies in this context. Finally, we provide a concise overview of the metrics used to evaluate segmentation quality.

### 3.1 Quantum computing principles

Quantum computing is founded on the principles of quantum mechanics, including qubits, superposition, and entanglement, which contrast sharply with classical binary computing. A qubit, unlike a classical bit, can be represented as a vector in a two-dimensional Hilbert space, existing in a coherent superposition of the basis states |0⟩ and |1⟩, expressed mathematically as |ψ⟩=α|0⟩+β|1⟩, where *α* and *β* are complex probability amplitudes satisfying ${\left|\alpha \right|}^{2}\;+\;{\left|\beta \right|}^{2}=1$. Entanglement, a non-classical correlation between qubits, enables the joint quantum state of multiple qubits to be non-separable, allowing for exponential increases in information encoding and computational parallelism. These quantum mechanical properties underpin quantum information theory and form the basis for quantum image representations, which exploit superposition and entanglement to encode, manipulate, and process image data in ways that are fundamentally different and potentially more efficient than classical pixel-based approaches.

### 3.2 Two-dimensional quantum image representation

In a gray-scale image with dimensions M×N, the intensity values are represented as f(m,n), where *m* and *n* denote the pixel coordinates. To capture local variations in intensity, the mean gray-level within a 3×3 window centered on each pixel (m,n) is computed. This local mean provides additional contextual information about the neighborhood of each pixel.

Using these intensity values and their corresponding local means, a two-dimensional (2D) histogram h(x,y) is constructed. The histogram combines the pixel intensities *x* with their computed local means *y* through empirical estimation. Represented as a matrix *H*, this 2D histogram encapsulates the joint distribution of pixel intensities and local means, offering a richer representation of the image by integrating individual pixel values with their surrounding context.

**Fig 1:**
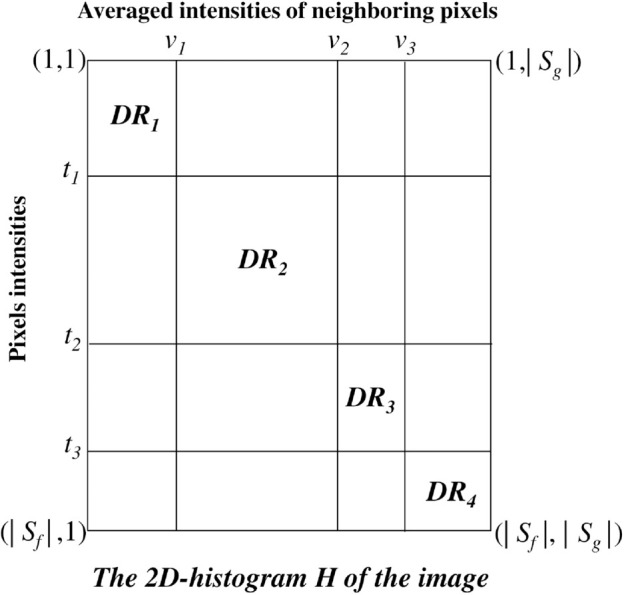
Illustration of the 2D histogram H partitioning using three thresholds. $\left|S_{f}\right|$ is the cardinality of the set of pixels intensities and $\left|S_{g}\right|$ is the cardinality of the set of averaged intensities of neighboring pixels.

The matrix *H* is further divided into diagonal rectangles, denoted as *DR*_*i*_, based on a set of predefined threshold values. These diagonal rectangles are treated as autonomous subsystems, where probabilities within each rectangle are normalized to ensure a coherent probabilistic interpretation. This partitioning approach enhances the granularity of the histogram representation, making it particularly useful for segmentation tasks. Fig 1 illustrates the partitioning of the 2D histogram *H* using three thresholding values, highlighting the structure of the obtained diagonal rectangles.

The flexible representation of quantum images (FRQI) allows the 2D histogram *H* to be conceptualized as an entangled state within a quantum system. In this framework, the quantum states are employed to encode the joint distribution of pixel intensities and their corresponding local means, leveraging the inherent correlations between these variables. This quantum encoding not only captures the statistical relationships but also integrates spatial information, providing a compact yet comprehensive representation of the image data.

The flexible representation of quantum images enables us to express the digital image as a quantum system [32,33]. Specifically, the 2D histogram *H* of the image can be encoded using the entangled state of a composite quantum system. For an image with 256 gray levels, we require up to 256×256 angles $\theta_{j}$ to encode the joint intensity distribution, according to the quantum superposition principle, as follows:

$$\left|I\left(\theta_{j}\right)\right\rangle =\cos\theta_{j}\left|0\right\rangle \;+\;\sin\theta_{j}\left|1\right\rangle ,$$ (1)

where $\theta_{j}=\frac{\pi }{2}\;w\left(x,y\right)$. Here, w(x,y)∈[0,1] denotes the normalized frequency of joint intensity (x,y), computed from the 2D histogram as:

$$w\left(x,y\right)=\frac{h\left(x,y\right)}{\sum_{x=0}^{255}\sum_{y=0}^{255}h\left(x,y\right)}.$$ (2)

The states |0⟩ and |1⟩ correspond to the quantum spin-down and spin-up states, respectively. This encoding ensures that the joint intensity information is fully captured in the quantum representation while respecting the constraints of quantum state amplitudes.

The quantum state of the subsystem *DR*_*i*_ is given by:

$$\left|I\left(DR_{i}\right)\right\rangle =\sum_{\left(x,y\right)\in DR_{i}}\left|I\left(\theta_{j}\right)\right\rangle .$$ (3)

Each diagonal rectangle *DR*_*i*_, derived from the partitioning of *H* using predefined threshold values, is treated as a distinct subsystem within the quantum state. The density operator $\rho_{i}$, represented mathematically by Eq (4), is used to characterize the quantum state associated with each *DR*_*i*_.

$$\rho_{i}=\left|I\left(DR_{i}\right)\right\rangle \left\langle I\left(DR_{i}\right)\right|=\left(\begin{array}{c} \sum_{\left(x,y\right)\in DR_{i}}\cos\theta_{j}\\ \sum_{\left(x,y\right)\in DR_{i}}\sin\theta_{j} \end{array}\right)\left(\begin{array}{cc} \sum_{\left(x,y\right)\in DR_{i}}\cos\theta_{j} & ,\sum_{\left(x,y\right)\in DR_{i}}\sin\theta_{j} \end{array}\right).$$ (4)

This operator encapsulates critical information about the joint probabilities and spatial relationships within the corresponding subsystem. By normalizing the probabilities within each *DR*_*i*_, the density operator ensures the probabilistic coherence required for quantum representation.

This entangled representation provides a powerful tool for capturing complex dependencies within the image. The quantum formalism inherently supports operations such as superposition and entanglement, allowing for more nuanced modeling of the spatial and statistical relationships within the image. As a result, this approach is particularly well-suited for applications such as image segmentation and analysis, where understanding local and global interactions is critical.

In this quantum framework, the diagonal rectangles are treated as autonomous quantum subsystems, each contributing to the overall quantum structure derived from the digital image. These subsystems interact to form a unified quantum setup, encapsulating the rich information content of the image. The quantum entropies, such as the Rényi or von Neumann entropies, can then be applied to quantify the amount of quantum information conveyed by the image. By analyzing these entropies, we can gain deeper insights into the distribution of information and the complexity of the image’s quantum state.

Rényi entropy is a generalized form of Shannon entropy that introduces a parameter allowing the adjustment of the sensitivity to different probability distributions. This flexibility makes it particularly suitable for multilevel thresholding tasks in image segmentation, where the goal is to optimally partition the image histogram into multiple regions. By tuning the Rényi parameter, the method can emphasize or de-emphasize certain pixel intensity distributions, improving the discrimination between regions of interest and background. Additionally, Rényi entropy’s mathematical properties facilitate efficient computation and robust segmentation, especially in complex images with varied textures and noise levels.

For the most up-to-date and detailed exploration of this quantum image representation, readers are encouraged to consult the work of Al-Mansor et al. [34], which provides a comprehensive treatment of this approach and its applications.

In terms of computational complexity, the quantum encoding of the 2D histogram *H*, based on the Flexible Representation of Quantum Images (FRQI), requires the computation of up to 256×256 angles $\theta_{j}$ for an 8-bit grayscale image. This corresponds to a total of $O\left(L^{2}\right)$ encoding operations, where *L* is the number of gray levels (typically 256). Each diagonal rectangle *DR*_*i*_ derived from the histogram is treated as a separate quantum subsystem, requiring individual construction of its quantum state and corresponding density matrix. The overall cost for constructing the entangled representation scales linearly with the number of such subsystems and quadratically with the image gray-level resolution.

The computational bottleneck primarily lies in the histogram construction and the quantum state preparation. However, these steps are performed once per image and can be parallelized, especially in classical simulation environments. Although current simulations are implemented classically, the quantum formalism remains efficient in terms of data representation, providing a compact model that scales well in terms of memory, especially for high-dimensional or multi-channel data.

As for scalability, the method remains tractable for images up to 512×512 pixels with 256 gray levels. For larger images or real-time applications, the encoding step can be optimized through subsampling, histogram binning, or block-wise processing. Furthermore, the modular nature of the diagonal rectangle decomposition allows future implementations on quantum hardware to treat each subsystem independently, which could benefit from parallel quantum circuits.

### 3.3 Thresholding problem formulation

The core objective behind the proposed method is to identify the optimal diagonal rectangles, represented as *DR*_*i*_, where *i* varies from 1 to k+1. This endeavor aims to yield effective image segmentation. These *DR*_*i*_ are contingent on a set of threshold values denoted as $X^{*}=\left(t_{1}^{*}<\ldots <t_{k}^{*}|v_{1}^{*}<\ldots <v_{k}^{*}\right)$, which maximize a fitness function grounded in entropy. The thresholds in the initial group, $t_{i}^{*}$ for i=1,…,k, exert their influence on the matrix *H*’s rows, while the thresholds in the subsequent group, $v_{i}^{*}$ for i=1,…,k, impact the matrix *H*’s columns. Fig 1 clearly illustrates the use of thresholds in partitioning the 2D histogram *H* into diagonal rectangles.

To quantify the information embedded within the image, both quantum and classical Rényi entropies are harnessed, with the classical approach facilitating comparative analysis. The necessity lies solely in the optimal threshold values $t_{1}^{*}<\ldots <t_{k}^{*}$ to partition the set of gray levels into k+1 coherent classes. For a more comprehensive understanding, readers are encouraged to refer to [34].

The construction of the processed image follows this procedure:

Let f(m,n), 1≤m≤M, 1≤n≤N, denote the pixel intensities of a gray-level image of size M×N

Let $t_{1}^{*}<\ldots <t_{k}^{*}$, the optimal thresholding values and set $t_{0}^{*}=0$ then $t_{k\;+\;1}^{*}=255$

   For i=0:k

     For m=1:M

       For n=1:N

       $A_{i\;+\;1}=Mean\left\{f\left(m,n\right)/t_{i}^{*}\leq f\left(m,n\right)\leq t_{i\;+\;1}^{*}\right\}$

       $f\left(m,n\right)⟵A_{i\;+\;1}$ when $t_{i}^{*}\leq f\left(m,n\right)\leq t_{i\;+\;1}^{*}$

       End

     End

End

The obtained f(m,n), 1≤m≤M, 1≤n≤N, gives the segmented image.

The quantum Rényi entropy, as defined in [36,37], provides a means to quantify the quantum information encapsulated within each diagonal rectangle *DR*_*i*_. It is expressed as follows:

$$QR_{\alpha }\left(DR_{i}\right)=\frac{1}{1-\alpha }\ln trace\left(\rho_{i}^{\alpha }\right),$$ (5)

where α∈R\{1}. The parameter *α* controls the order of the entropy and adjusts the sensitivity of the measure to different probability distributions: lower values of *α* emphasize contributions from more probable events, while higher values give more weight to less probable (i.e., rare) events.

The cumulative quantum information of the image is subsequently determined as:

$$QR_{\alpha }\left(H\right)=\sum_{i=1}^{k\;+\;1}QR_{\alpha }\left(DR_{i}\right).$$ (6)

Ultimately, the vector comprising the optimal threshold values $X^{*}=\left(t_{1}^{*}<\ldots <t_{k}^{*}|v_{1}^{*}<\ldots <v_{k}^{*}\right)$ is derived through the maximization of the overall entropy as referenced in (6). This is expressed as follows:

$$X^{*}=\underset{X\in \left(S_{f}^{k}\;\times \;S_{g}^{k}\right)}{Arg\max}QR_{\alpha }\left(H\right),$$ (7)

where *S*_*f*_ denotes the set of pixels intensities and *S*_*g*_ denotes the set of averaged intensities of neighboring pixels as illustrated in Fig 1.

Within the classical framework, the quantity of information harbored within each diagonal rectangle *DR*_*i*_ will be measured by the classical Rényi entropy [37], namely

$$CR_{\alpha }\left(DR_{i}\right)=\frac{1}{1-\alpha }\ln\sum_{\left(x,y\right)\in DR_{i}}w{\left(x,y\right)}^{\alpha }.$$ (8)

The complete entropy of the image is subsequently determined as:

$$CR_{\alpha }\left(H\right)=\sum_{i=1}^{k\;+\;1}CR_{\alpha }\left(DR_{i}\right),$$ (9)

Like in (7), the optimal thresholding values are obtained by solving the following problem:

$$X^{*}=\underset{X\in \left(S_{f}^{k}\;\times \;S_{g}^{k}\right)}{Arg\max}CR_{\alpha }\left(H\right).$$ (10)

The two-dimensional quantum Rényi entropy offers a distinct and complementary perspective on the information content of a quantum state, contrasting with the more widely known von Neumann entropy. As a powerful tool in quantum information theory, it provides deeper insights into the underlying structure and distribution of information within quantum states. Unlike the standard von Neumann entropy, which captures only the uncertainty or disorder in a system, the quantum Rényi entropy incorporates a broader range of information metrics, allowing for a more nuanced analysis of quantum states. This makes it particularly useful in contexts where understanding the complexity and finer details of quantum correlations is essential, enabling more refined interpretations and applications in quantum computing, quantum cryptography, and other advanced quantum technologies.

### 3.4 Simplified workflow for the proposed method

To better illustrate the proposed segmentation approach, consider a simplified example using a small 8×8 grayscale image patch. For each pixel, the local mean intensity within a 3×3 window is computed. For border pixels, this mean is calculated using only the neighboring pixels that lie within the boundaries of the patch. As a result, we obtain 64 intensity–mean pairs and thus 64 angles $\theta_{j}$. These pairs are used to construct the 2D histogram, which is then partitioned into diagonal rectangles based on preliminary threshold values. For each diagonal subsystem, the quantum state is encoded via angles $\theta_{j}$ as described previously. The Rényi entropy is calculated over these subsystems to quantify information content. Using Particle Swarm Optimization, candidate thresholds are iteratively updated to maximize the overall entropy, resulting in an optimal segmentation. This step-by-step example demonstrates the practical workflow of the method, clarifying its computational steps and optimization process. Moreover, Sects 3.1 and 3.2 along with Fig 1 provide detailed illustrations of the different stages involved in the proposed segmentation method.

**Fig 2:**
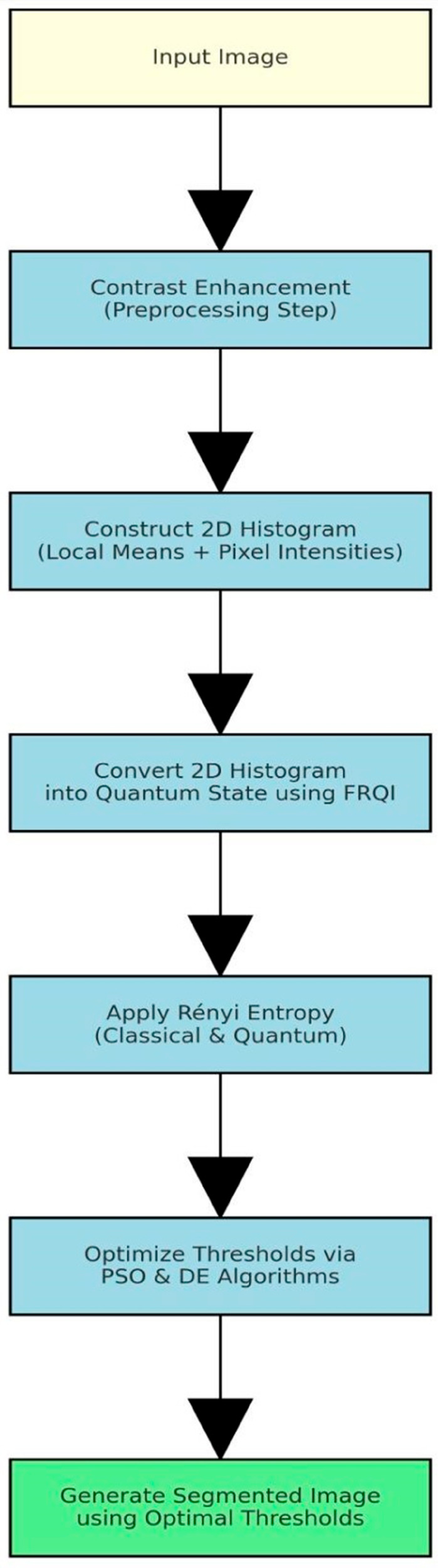
Flowchart of proposed quantum-based image segmentation procedure.

Fig 2 presents the flowchart of the proposed quantum-based image segmentation procedure. The image contrast is enhanced before being segmented.

Clearly, solving the combinatorial optimization problems (7) and (10) in a deterministic manner is not feasible due to their inherent complexity. In this study, we have chosen to employ two well-established metaheuristic algorithms, namely Particle Swarm Optimization (PSO) and Differential Evolution (DE), to efficiently tackle these problems. The selection of PSO and DE is driven by their proven effectiveness and widespread application across a variety of domains, where they have demonstrated remarkable performance in solving complex optimization challenges.

Particle Swarm Optimization (PSO) is a population-based stochastic optimization technique inspired by the social behavior of bird flocks or fish schools. In PSO, a swarm of candidate solutions, called particles, explores the search space by updating their positions and velocities based on their own experience and that of their neighbors. This cooperative behavior enables the swarm to efficiently converge towards optimal or near-optimal solutions. PSO is particularly well-suited for threshold optimization in image segmentation due to its ability to handle complex, multi-dimensional search spaces and find global optima without requiring gradient information.

Differential Evolution (DE) is an evolutionary optimization algorithm that iteratively improves candidate solutions using operations inspired by natural selection, mutation, and recombination. Starting from a randomly initialized population, DE generates new candidate solutions by combining existing ones and selects those with better fitness values for the next generation. Its simplicity, robustness, and capability to efficiently explore complex and nonlinear search spaces make DE particularly effective for optimizing threshold values in image segmentation.

### 3.5 Segmentation quality metrics

To rigorously assess the effectiveness of the proposed segmentation methods, we employ a set of nine well-established and complementary image quality metrics. These metrics have been carefully selected to capture various aspects critical to multilevel grayscale image segmentation, including contrast enhancement, structural fidelity, information preservation, and perceptual quality.

- PSNR (Peak Signal-to-Noise Ratio) quantifies the fidelity of the processed image compared to the original. Although it does not align perfectly with human perception, it remains a standard reference for measuring global distortion, particularly useful in benchmarking.
- SSIM (Structural Similarity Index) provides a perceptually meaningful measure by evaluating luminance, contrast, and structure similarities. It complements PSNR by emphasizing how humans perceive visual quality.
- AMBE (Absolute Mean Brightness Error) evaluates the brightness preservation capability, which is crucial in applications where natural brightness needs to be maintained after segmentation.
- CII (Contrast Improvement Index) and CIR (Contrast Improvement Ratio) directly assess the degree of contrast enhancement introduced by the segmentation. These are particularly relevant since one of the primary goals of the proposed method is to enhance visual separability between segmented regions.
- SD (Standard Deviation) serves as a simple but effective indicator of image contrast and texture variability. A higher SD suggests that the segmentation has introduced meaningful variance between regions.
- DE (Edge Density) quantifies the amount of edge information in the segmented image. Since effective segmentation should preserve or emphasize boundaries, this metric helps assess detail retention.
- REC (Relative Entropy Change) captures variations in information content between the original and segmented images. It is especially useful for evaluating the transformation’s impact on the underlying image complexity.
- SF (Spatial Frequency) measures the level of detail and texture, providing an objective assessment of the image’s spatial richness after segmentation.

All metrics except AMBE are positively correlated with quality—higher values indicate better results. The joint use of these metrics ensures that the evaluation covers both objective fidelity (PSNR, AMBE), perceptual quality (SSIM), and segmentation-specific attributes (CII, CIR, DE, REC, SF). Their combined use strengthens the reliability of the comparative analysis and aligns directly with the multi-criteria objectives of our segmentation task.

Further mathematical definitions and benchmarking roles of these metrics are detailed in [38].

## 4 Experimental results and discussion

To evaluate the performance and robustness of the proposed segmentation framework, we selected a diverse set of twenty widely recognized grayscale benchmark images commonly used in the image processing community. This collection encompasses classical test images such as Lena, Cameraman, Peppers, Barbara, Boats, and Mandrill, which exhibit a variety of visual characteristics including smooth transitions, soft and intricate textures, complex patterns, and sharp edges. The image dimensions range from 256×256 to 512×512 pixels. This carefully curated dataset ensures coverage of diverse spatial frequencies, contrast levels, noise content, and structural complexities, providing a comprehensive and balanced evaluation of the method’s robustness and generalizability.

For each benchmark image, segmentation was performed and the resulting output was quantitatively assessed using standardized procedures. Metric values—including PSNR, SSIM, and others—were computed on the normalized 8-bit grayscale images to maintain consistency.

In accordance with copyright requirements, we only displayed segmentation results for two representative images (Tiger and Eyes) that are released under the Creative Commons Attribution (CC BY 4.0) license.

The goal of the experiments is twofold: to compare the two optimizers DE and PSO and to assess the effectiveness of the quantum two-dimensional multilevel thresholding approach. In total, four segmentation methods will be compared in this Section, namely, DE-CR (to designate: Differential Evolution with Classical Rényi entropy), PSO-CR (to designate: Particle Swarm Optimization with Classical Rényi entropy), DE-QR (to designate: Differential Evolution with Quantum Rényi entropy) and PSO-QR (to designate: Particle Swarm Optimization with Quantum Rényi entropy).

The numerical experiments are performed using MATLAB, the version R2021a. The maximal number of iterations was set to 1000 and the population size to 30 for both, DE and PSO algorithms. The DE algorithm is configured with a mutation factor *F* = 0.8 and a crossover rate *CR* = 0.9. For PSO, the swarm size is set to 30 particles, cognitive and social coefficients $c_{1}=c_{2}=2$, and the inertia weight decreases linearly from 0.9 to 0.4 over iterations. These parameter choices balance exploration and exploitation to promote efficient convergence.

The number of thresholding values was gradually taken equal to 2, 4 then 7 to observe the convergence and the complexity evolution of the competing methods. Finally, considering the results of Ben Ishak [39], the R ényi’s parameter *α* was set to 0.01.

Prior to applying the four image segmentation methods, we used a recent contrast enhancement technique introduced in [38]. To choose this method, we carried out preliminary experiments comparing it with two other approaches developed in [40] and [41], respectively. These preliminary experiments revealed that the segmentation results obtained after applying contrast enhancement were significantly improved. This improvement can be attributed to the fact that contrast enhancement markedly improves the distribution of gray levels in the images, which is a crucial factor in the calculation of information via entropy-based functions. Enhanced gray-level distributions lead to more distinct and separable regions, thereby facilitating more accurate and reliable thresholding.

**Fig 3:**
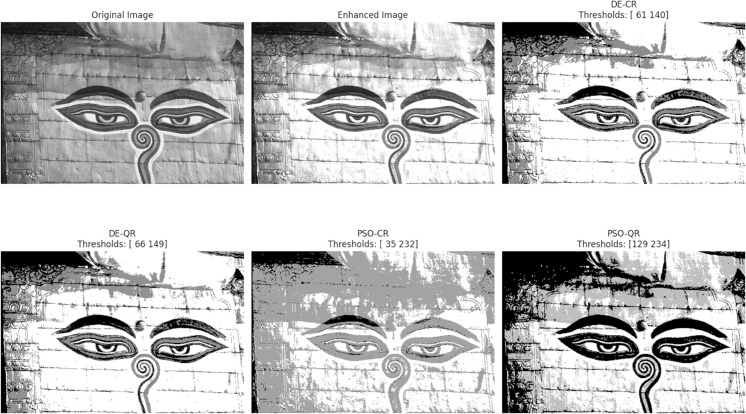
An example of the segmentation results based on 2 thresholds. The optimal thresholding values are displayed above each processed image.

Figs 3 through 8 illustrate the segmentation results obtained by various competing methods on two sample images, providing a visual comparison. The image displayed right next to the original corresponds to the contrast-enhanced version, which is then subjected to segmentation. We experimented with 2, 4, and 7 thresholds, and the optimal threshold values are clearly indicated above each segmented image. These thresholds were used to partition the image pixels into homogeneous regions based on their gray-level distribution.

**Fig 4:**
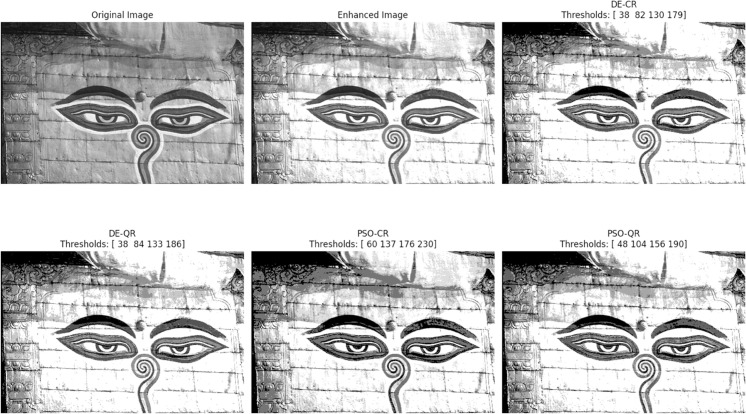
An example of the segmentation results based on 4 thresholds. The optimal thresholding values are displayed above each processed image.

**Fig 5:**
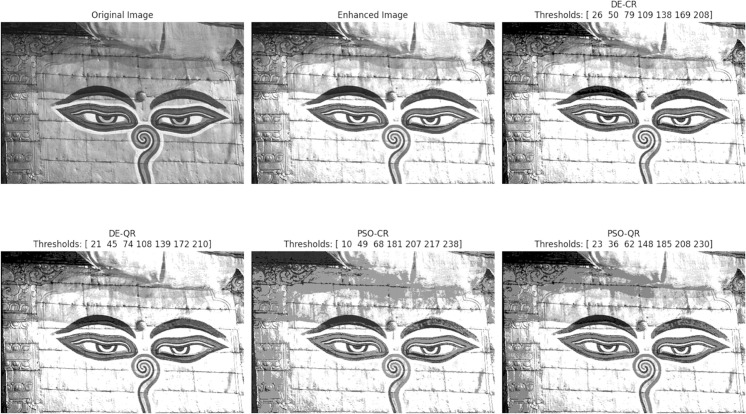
An example of the segmentation results based on 7 thresholds. The optimal thresholding values are displayed above each processed image.

**Fig 6:**
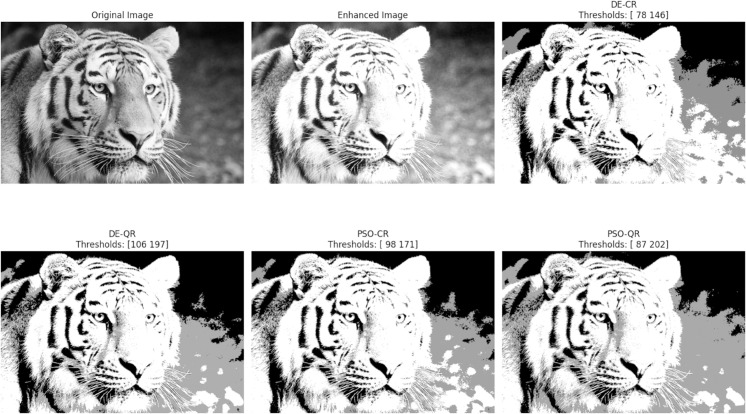
An example of the segmentation results based on 2 thresholds. The optimal thresholding values are displayed above each processed image.

**Fig 7:**
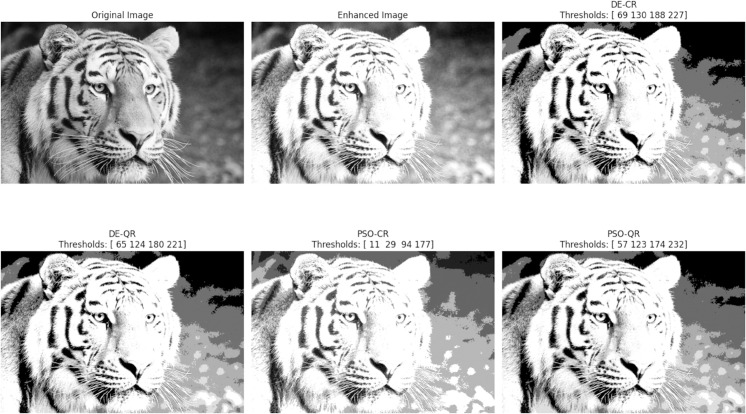
An example of the segmentation results based on 4 thresholds. The optimal thresholding values are displayed above each processed image.

**Fig 8:**
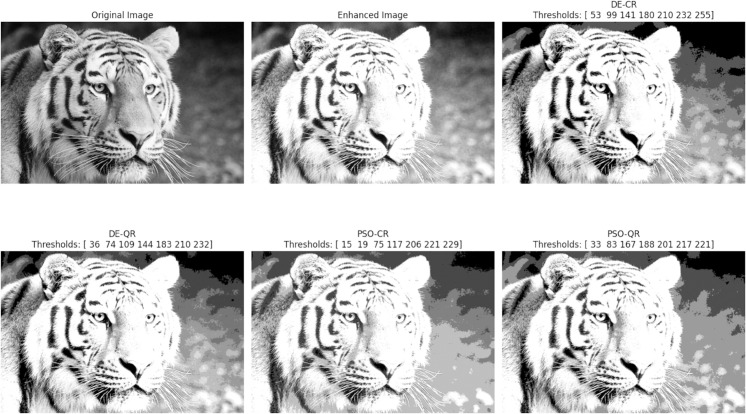
An example of the segmentation results based on 7 thresholds. The optimal thresholding values are displayed above each processed image.

To provide an intuitive understanding of the segmentation results, we present selected images illustrating the visual quality achieved by the different methods. The preliminary contrast enhancement step positively impacts segmentation quality by increasing the separability of regions, facilitating more accurate threshold determination. While the use of gray and dark gray tones against a gray background can make visual distinction between regions challenging, PSO-QR demonstrates superior capability in delineating subtle intensity differences, particularly in low-contrast areas. This advantage is due to the effective optimization of quantum Ré nyi entropy, which better captures the image’s structural details.

Furthermore, as the number of thresholds increases, the segmented images produced by PSO-QR progressively approximate their contrast-enhanced counterparts, reflecting improved segmentation precision. These qualitative observations complement the quantitative assessment based on nine segmentation quality metrics. The detailed results, summarized through boxplots and statistical tables, constitute the primary and most reliable foundation for the comparative analysis and conclusions of this study.

**Fig 9:**
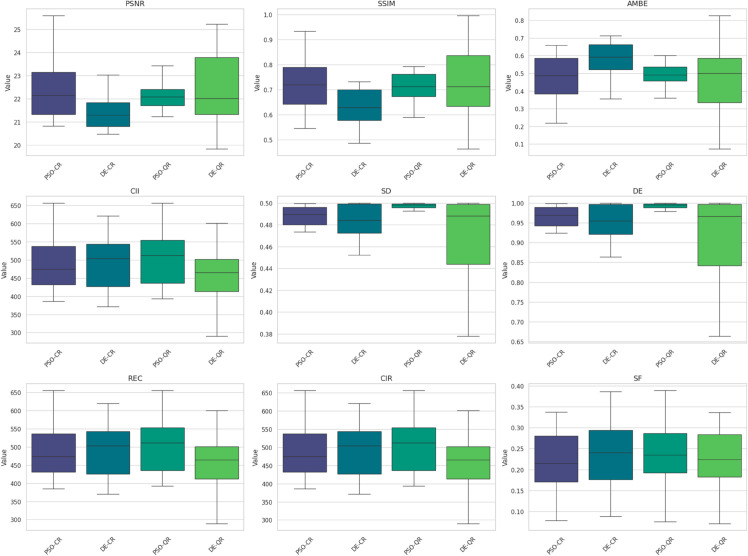
Boxplots of segmentation evaluation metrics when using 2 thresholds.

**Fig 10:**
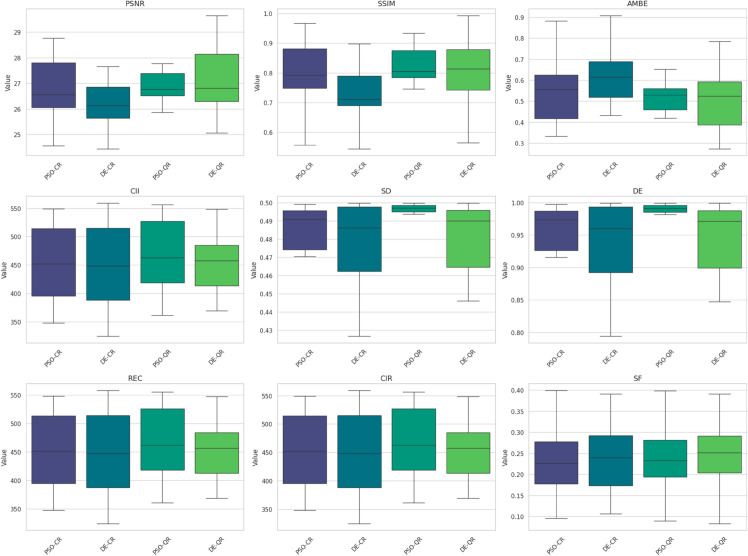
Boxplots of segmentation evaluation metrics when using 4 thresholds.

**Fig 11:**
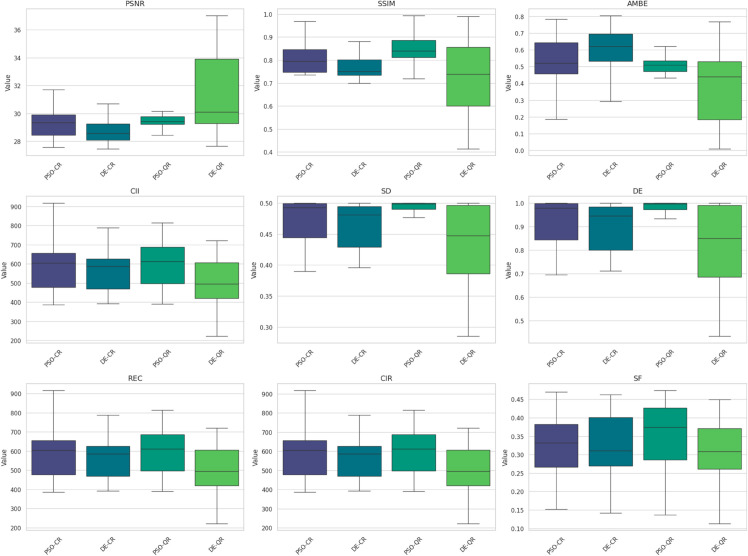
Boxplots of segmentation evaluation metrics when using 7 thresholds.

Figs 9 to 11 present the box plots of the segmentation evaluation metrics (PSNR, SSIM, SF, CII, DE, and REC) for the four competing methods across twenty test images, with the number of thresholds *k* set to 2, 4, and 7, respectively.

A consistent upward trend is observed for PSNR and SSIM values across all methods as *k* increases. For instance, in Fig 9 (with *k* = 2), PSNR values show notable variability and generally lower medians compared to those in Fig 11 (with *k* = 7), where PSNR distributions are shifted upwards with tighter interquartile ranges. This indicates that the segmented images better approximate the original image when more thresholds are used. A similar pattern is visible for SSIM, suggesting that multilevel segmentation enhances structural fidelity. While the improvements in other metrics (such as SF, CII, DE, and REC) are less pronounced, they tend to follow the same trend, particularly between *k* = 2 and *k* = 7. These observations provide quantitative support for the known advantage of multilevel thresholding in capturing fine image details, even though it introduces greater computational complexity.

Comparative inspection of the box plots across all values of *k* also shows that the PSO-QR method consistently yields superior median scores in PSNR and SSIM, while achieving competitive or best values in the remaining metrics. For example, in Fig 10 (with *k* = 4), PSO-QR presents both higher central tendency and narrower dispersion in SSIM and PSNR than the other methods, implying more accurate and stable segmentation outcomes. This trend continues in Fig 11 (with *k* = 7), where PSO-QR maintains leading performance across most metrics. Furthermore, the box lengths (representing variance) are generally shorter for PSO-QR, particularly in the SSIM and DE metrics, indicating greater consistency across different images.

Overall, these empirical results validate the benefits of using a multilevel segmentation scheme and suggest that the PSO-QR method is not only effective in optimizing segmentation quality but also robust across varying threshold counts.

Table 1 presents the ranking of the four segmentation methods across each evaluation metric, based on the box plots shown in Figs 9 through 11. The best-performing method is assigned rank 1 according to the average value of the respective metric. In the event of a tie, the method with the lowest variance is ranked higher. Conversely, the method with the poorest performance receives rank 4. For each segmentation method, the overall ranking (OR) is computed as the sum of its ranks across the nine metrics. A lower OR indicates better overall performance and is highlighted in bold within the table.

**Table 1:** Ranking of segmentation methods based on the nine evaluation metrics. OR is the overall ranking.

	Methods//Metrics	PSNR	SSIM	AMBE	CII	SD	DE	REC	CIR	SF	OR
*k* = 2	PSO-CR	1	1	2	3	2	2	3	3	4	21
	DE-CR	4	4	4	2	4	4	2	2	1	27
	PSO-QR	2	2	1	1	1	1	1	1	2	**12**
	DE-QR	3	3	3	4	3	3	4	4	3	30
*k* = 4	PSO-CR	3	3	3	3	2	2	3	3	4	26
	DE-CR	4	4	4	4	4	4	4	4	2	34
	PSO-QR	2	2	2	1	1	1	1	1	3	**14**
	DE-QR	1	1	1	2	3	3	2	2	1	16
*k* = 7	PSO-CR	3	2	3	2	2	2	2	2	2	20
	DE-CR	4	3	4	3	3	3	3	3	3	29
	PSO-QR	2	1	2	1	1	1	1	1	1	**11**
	DE-QR	1	4	1	4	4	4	4	4	4	30

The results provided by Table 1 clearly demonstrate the superiority of the PSO-QR method, which consistently achieves the lowest OR values for all tested values of *k* (the number of thresholds). For instance, at *k* = 7, PSO-QR attains an OR of 11, significantly outperforming competing methods such as PSO-CR (OR = 20), DE-QR (OR = 30), and DE-CR (OR = 29), underscoring its robust and consistent performance.

Moreover, the DE-QR method ranks second at *k* = 4 with an OR of 16, while PSO-CR holds the second position at *k* = 2 and *k* = 7, with OR values of 21 and 20, respectively. In contrast, DE-CR consistently ranks third or fourth, with OR values ranging from 27 to 34 across scenarios, reflecting its comparatively weaker performance.

These observations suggest that the PSO algorithm is particularly effective in handling a large number of thresholds, thereby optimizing multilevel segmentation. Meanwhile, the QR method enhances the extraction of information from the two-dimensional intensity distribution of the image, contributing to improved segmentation quality. The synergy of these two features positions PSO-QR as the top-performing method, well ahead of competing approaches.

The Wilcoxon Signed Rank Test is employed to analyze paired observations, where each pair corresponds to a direct comparison between the segmentation methods across each evaluation metric. As a non-parametric test, it determines whether the observed differences between methods are statistically significant without assuming normality, offering a robust insight into the comparative behavior of the algorithms under consideration [38,42].

Tables 2 to 4 display the test results for threshold levels *k* = 2, 4, and 7. The null hypothesis assumes that the paired samples originate from the same distribution. The test outputs a W statistic and an associated p-value: the smaller the p-value, the stronger the evidence against the null hypothesis.

**Table 2:** Wilcoxon signed test for the nine metrics when using 2 thresholds. The p-value is provided in parentheses under the test statistic W.

Methods//Metrics	PSNR	SSIM	AMBE	CII	SD	DE	REC	CIR	SF
DE-CR/PSO-QR	$\underset{\left(<0.001\right)}{1}$	$\underset{\left(<0.001\right)}{1}$	$\underset{\left(<0.001\right)}{1}$	$\underset{\left(0.007\right)}{35}$	$\underset{\left(0.006\right)}{34}$	$\underset{\left(0.006\right)}{34}$	$\underset{\left(0.007\right)}{35}$	$\underset{\left(0.007\right)}{35}$	$\underset{\left(0.701\right)}{94}$
DE-CR/DE-QR	$\underset{\left(<0.001\right)}{12}$	$\underset{\left(<0.001\right)}{12}$	$\underset{\left(<0.001\right)}{13}$	$\underset{\left(0.354\right)}{72}$	$\underset{\left(0.375\right)}{73}$	$\underset{\left(0.375\right)}{73}$	$\underset{\left(0.354\right)}{72}$	$\underset{\left(0.354\right)}{72}$	$\underset{\left(0.334\right)}{71}$
PSO-QR/DE-QR	$\underset{\left(0.294\right)}{76}$	$\underset{\left(0.348\right)}{79}$	$\underset{\left(0.329\right)}{78}$	$\underset{\left(0.004\right)}{32}$	$\underset{\left(0.004\right)}{31}$	$\underset{\left(0.004\right)}{31}$	$\underset{\left(0.004\right)}{32}$	$\underset{\left(0.004\right)}{32}$	$\underset{\left(0.245\right)}{73}$
PSO-CR/DE-CR	$\underset{\left(<0.001\right)}{0}$	$\underset{\left(<0.001\right)}{0}$	$\underset{\left(<0.001\right)}{0}$	$\underset{\left(0.621\right)}{91}$	$\underset{\left(0.545\right)}{88}$	$\underset{\left(0.545\right)}{88}$	$\underset{\left(0.621\right)}{91}$	$\underset{\left(0.621\right)}{91}$	$\underset{\left(0.277\right)}{75}$
PSO-CR/PSO-QR	$\underset{\left(0.521\right)}{87}$	$\underset{\left(0.595\right)}{90}$	$\underset{\left(0.840\right)}{99}$	$\underset{\left(<0.001\right)}{0}$	$\underset{\left(<0.001\right)}{0}$	$\underset{\left(<0.001\right)}{0}$	$\underset{\left(<0.001\right)}{0}$	$\underset{\left(<0.001\right)}{0}$	$\underset{\left(0.029\right)}{47}$
PSO-CR/DE-QR	$\underset{\left(0.444\right)}{76}$	$\underset{\left(0.573\right)}{81}$	$\underset{\left(0.375\right)}{73}$	$\underset{\left(0.629\right)}{83}$	$\underset{\left(0.658\right)}{84}$	$\underset{\left(0.658\right)}{84}$	$\underset{\left(0.629\right)}{83}$	$\underset{\left(0.629\right)}{83}$	$\underset{\left(0.717\right)}{86}$

**Table 3:** Wilcoxon signed test for the nine metrics when using 4 thresholds. The p-value is provided in parentheses under the test statistic W.

Methods//Metrics	PSNR	SSIM	AMBE	CII	SD	DE	REC	CIR	SF
DE-CR/PSO-QR	$\underset{\left(<0.001\right)}{0}$	$\underset{\left(<0.001\right)}{0}$	$\underset{\left(<0.001\right)}{0}$	$\underset{\left(0.002\right)}{19}$	$\underset{\left(0.002\right)}{19}$	$\underset{\left(0.002\right)}{19}$	$\underset{\left(0.002\right)}{19}$	$\underset{\left(0.002\right)}{19}$	$\underset{\left(0.967\right)}{94}$
DE-CR/DE-QR	$\underset{\left(<0.001\right)}{18}$	$\underset{\left(<0.001\right)}{15}$	$\underset{\left(<0.001\right)}{16}$	$\underset{\left(0.927\right)}{102}$	$\underset{\left(0.784\right)}{97}$	$\underset{\left(0.784\right)}{97}$	$\underset{\left(0.927\right)}{102}$	$\underset{\left(0.927\right)}{102}$	$\underset{\left(0.898\right)}{101}$
PSO-QR/DE-QR	$\underset{\left(0.521\right)}{87}$	$\underset{\left(0.545\right)}{88}$	$\underset{\left(0.701\right)}{94}$	$\underset{\left(<0.001\right)}{19}$	$\underset{\left(0.001\right)}{22}$	$\underset{\left(0.001\right)}{22}$	$\underset{\left(<0.001\right)}{19}$	$\underset{\left(<0.001\right)}{19}$	$\underset{\left(0.430\right)}{83}$
PSO-CR/DE-CR	$\underset{\left(<0.001\right)}{0}$	$\underset{\left(<0.001\right)}{0}$	$\underset{\left(<0.001\right)}{0}$	$\underset{\left(0.105\right)}{61}$	$\underset{\left(0.063\right)}{55}$	$\underset{\left(0.063\right)}{55}$	$\underset{\left(0.105\right)}{61}$	$\underset{\left(0.105\right)}{61}$	$\underset{\left(0.348\right)}{79}$
PSO-CR/PSO-QR	$\underset{\left(0.701\right)}{94}$	$\underset{\left(0.521\right)}{87}$	$\underset{\left(0.474\right)}{85}$	$\underset{\left(<0.001\right)}{0}$	$\underset{\left(<0.001\right)}{0}$	$\underset{\left(<0.001\right)}{0}$	$\underset{\left(<0.001\right)}{0}$	$\underset{\left(<0.001\right)}{0}$	$\underset{\left(0.277\right)}{75}$
PSO-CR/DE-QR	$\underset{\left(0.082\right)}{58}$	$\underset{\left(0.189\right)}{69}$	$\underset{\left(0.113\right)}{62}$	$\underset{\left(0.927\right)}{102}$	$\underset{\left(0.956\right)}{103}$	$\underset{\left(0.956\right)}{103}$	$\underset{\left(0.927\right)}{102}$	$\underset{\left(0.927\right)}{102}$	$\underset{\left(0.164\right)}{67}$

**Table 4:** Wilcoxon signed test for the nine metrics when using 7 thresholds. The p-value is provided in parentheses under the test statistic W.

Methods//Metrics	PSNR	SSIM	AMBE	CII	SD	DE	REC	CIR	SF
DE-CR/PSO-QR	$\underset{\left(0.001\right)}{20}$	$\underset{\left(0.001\right)}{20}$	$\underset{\left(<0.001\right)}{18}$	$\underset{\left(<0.001\right)}{12}$	$\underset{\left(<0.001\right)}{13}$	$\underset{\left(<0.001\right)}{13}$	$\underset{\left(<0.001\right)}{12}$	$\underset{\left(<0.001\right)}{12}$	$\underset{\left(0.044\right)}{45}$
DE-CR/DE-QR	$\underset{\left(0.001\right)}{21}$	$\underset{\left(<0.001\right)}{12}$	$\underset{\left(0.001\right)}{20}$	$\underset{\left(0.418\right)}{74}$	$\underset{\left(0.373\right)}{72}$	$\underset{\left(0.373\right)}{72}$	$\underset{\left(0.418\right)}{74}$	$\underset{\left(0.418\right)}{74}$	$\underset{\left(0.123\right)}{56}$
PSO-QR/DE-QR	$\underset{\left(0.170\right)}{54}$	$\underset{\left(0.170\right)}{54}$	$\underset{\left(0.184\right)}{55}$	$\underset{\left(0.002\right)}{17}$	$\underset{\left(0.002\right)}{17}$	$\underset{\left(0.002\right)}{17}$	$\underset{\left(0.002\right)}{17}$	$\underset{\left(0.002\right)}{17}$	$\underset{\left(0.009\right)}{26}$
PSO-CR/DE-CR	$\underset{\left(<0.001\right)}{0}$	$\underset{\left(<0.001\right)}{0}$	$\underset{\left(<0.001\right)}{0}$	$\underset{\left(0.060\right)}{48}$	$\underset{\left(0.133\right)}{57}$	$\underset{\left(0.123\right)}{56}$	$\underset{\left(0.060\right)}{48}$	$\underset{\left(0.060\right)}{48}$	$\underset{\left(0.352\right)}{71}$
PSO-CR/PSO-QR	$\underset{\left(0.306\right)}{62}$	$\underset{\left(0.285\right)}{61}$	$\underset{\left(0.156\right)}{53}$	$\underset{\left(<0.001\right)}{1}$	$\underset{\left(<0.001\right)}{1}$	$\underset{\left(<0.001\right)}{1}$	$\underset{\left(<0.001\right)}{1}$	$\underset{\left(<0.001\right)}{1}$	$\underset{\left(0.052\right)}{41}$
PSO-CR/DE-QR	$\underset{\left(0.352\right)}{71}$	$\underset{\left(0.332\right)}{70}$	$\underset{\left(0.257\right)}{66}$	$\underset{\left(0.144\right)}{58}$	$\underset{\left(0.168\right)}{60}$	$\underset{\left(0.181\right)}{61}$	$\underset{\left(0.144\right)}{58}$	$\underset{\left(0.144\right)}{58}$	$\underset{\left(0.241\right)}{65}$

The analysis of the results reveals several key patterns:

- For *k* = 2, the Wilcoxon test indicates statistically significant differences between DE-CR and PSO-QR in 8 out of 9 metrics (all metrics except SF), with p-values close to zero (*W* = 1 for PSNR, SSIM, and AMBE; W=34−35 for the rest). This highlights a clear performance gap between these two methods. Similarly, PSO-QR vs. PSO-CR shows significant differences in 6 out of 9 metrics (e.g., *W* = 0 for CII, SD, DE, REC, CIR; *W* = 47 for SF), confirming PSO-QR’s superiority. In contrast, metrics such as SF (*W* = 94 for DE-CR vs PSO-QR) appear less discriminative at this threshold level.
- For *k* = 4, the results maintain the same trend. PSO-QR outperforms DE-CR significantly on 8 out of 9 metrics, with *W* = 0 for PSNR, SSIM, and AMBE, and *W* = 19 for the rest (except SF where *W* = 94). Notably, the PSO-QR vs. DE-QR comparison also yields *W* values as low as 19–22 in 6 out of 9 metrics, indicating that PSO-QR remains statistically stronger. The PSO-CR vs. DE-CR pair shows no significant difference in PSNR, SSIM, and AMBE (*W* = 0 ), but higher *W* values for other metrics (e.g., *W* = 79 for SF), suggesting similar behavior in intensity preservation but divergence in spatial and structural fidelity.
- For *k* = 7, while the magnitude of difference slightly decreases, PSO-QR still shows statistically significant differences in 7 out of 9 metrics when compared with DE-CR, with *W* values between 12 and 20. Notably, the differences disappear for CIR and SF (*W* = 45). The PSO-CR vs. PSO-QR comparison yields *W* = 1 or lower in 6 metrics (CII, SD, DE, REC, CIR, SF), confirming PSO-QR’s clear edge, especially in spatial and edge-based metrics.

Overall, the Wilcoxon test results substantiate the observed performance gaps among the segmentation methods and reinforce the superiority and robustness of PSO-QR, particularly as the number of thresholds increases. Across all threshold levels, PSO-QR consistently demonstrates statistically significant improvements in most metrics compared to DE-CR and PSO-CR, and maintains an advantage over DE-QR in several key metrics. This robustness across varying segmentation complexity levels (*k*) confirms PSO-QR’s adaptability and effectiveness, validating its top position identified in the ranking analysis (Table 1). Furthermore, these findings underscore the necessity of choosing evaluation metrics carefully, as certain metrics like CIR and SF tend to show fewer discriminative differences, while PSNR, SSIM, and AMBE offer clearer distinctions among methods.

Finally, Table 5 presents the average number of iterations (N), execution time in seconds (T), and maximum value of the fitness function (Y) obtained during the entropy-based optimization process, for each of the four segmentation methods. These averages were computed over a set of twenty grayscale images, for three threshold levels: *k* = 2, 4, and 7. The best-performing results in each category are highlighted in bold.

**Table 5:** Comparison of DE and PSO optimization performance when maximizing CR and QR entropies. N is the number of iterations, T is the execution time and Y is the reached optimum.

	k=2			k=4			k=7		
	N	T	Y	N	T	Y	N	T	Y
DE-CR	256.63	36.45	24.87	630.22	95.67	37.56	804.53	135.09	51.36
PSO-CR	**49.22**	**7.52**	**25.07**	**91.08**	**14.82**	**38.31**	**235.52**	**42.11**	**52.83**
.25em0em0em DE-QR	135.98	17.55	2.41	414.33	54.20	3.84	689.56	108.67	6.22
PSO-QR	**47.88**	**6.23**	**2.83**	**82.09**	**11.43**	**4.11**	**215.21**	**36.13**	**6.81**

The data clearly demonstrate that PSO-based methods outperform their DE-based counterparts across all values of *k*, especially as the segmentation task becomes more complex. For instance, at *k* = 2, the optimum entropy values (Y) are nearly identical for CR-based methods (PSO-CR: 25.07 vs. DE-CR: 24.87), and also very close for QR-based methods (PSO-QR: 2.83 vs. DE-QR: 2.41). However, PSO achieves these optima much more efficiently, requiring fewer iterations (PSO-QR: 47.88 vs. DE-QR: 135.98; PSO-CR: 49.22 vs. DE-CR: 256.63) and considerably less execution time (PSO-QR: 6.23s vs. DE-QR: 17.55s; PSO-CR: 7.52s vs. DE-CR: 36.45s).

This performance gap becomes even more pronounced at higher threshold levels. For *k* = 4, PSO-QR reaches an entropy value of 4.11 in just 82.09 iterations and 11.43 seconds, compared to DE-QR which achieves a slightly lower optimum (3.84) with 414.33 iterations and 54.20 seconds. Likewise, PSO-CR achieves 38.31 in 91.08 iterations and 14.82 seconds, while DE-CR requires 630.22 iterations and 95.67 seconds to reach a lower value of 37.56.

At *k* = 7, where the optimization landscape is more complex, the advantages of PSO are most evident. PSO-QR achieves the best overall entropy (6.81) in only 215.21 iterations and 36.13 seconds, while DE-QR lags behind with 6.22 despite 689.56 iterations and 108.67 seconds. Similarly, PSO-CR reaches 52.83 in 235.52 iterations and 42.11 seconds, outperforming DE-CR, which requires 804.53 iterations and 135.09 seconds to reach 51.36.

These results confirm that PSO not only converges faster but also reaches better or equal solutions compared to DE, and that this difference amplifies as k increases, i.e., when the optimization problem becomes more challenging due to the increased dimensionality of the search space.

Additionally, the comparison between CR and QR entropy formulations reveals a consistent advantage for QR in terms of computational efficiency. Across all values of k, QR-based methods require fewer iterations and less time than their CR-based counterparts. For example, at k = 7, PSO-QR converges in 215.21 iterations versus 235.52 for PSO-CR, and executes in 36.13 seconds versus 42.11. This trend underscores the practical benefit of using the QR entropy, especially in time-sensitive applications.

**Fig 12:**
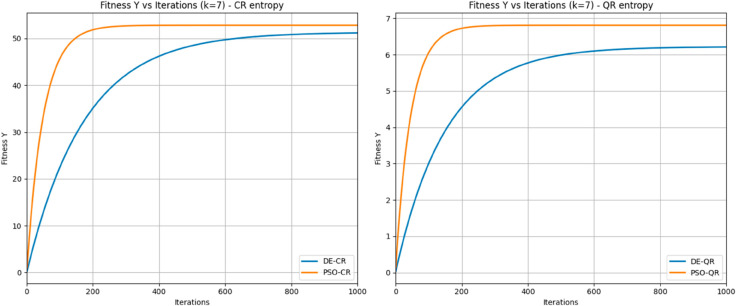
Comparison of DE and PSO fitness convergence for CR and QR entropies at *k* = 7.

Fig 12 shows fitness Y convergence versus iterations for *k* = 7, comparing DE and PSO under CR (left) and QR (right) entropies. PSO consistently converges faster and reaches slightly better fitness values than DE. Specifically, PSO-CR and PSO-QR reach their optima near 230 and 210 iterations, respectively, while DE-CR and DE-QR take about 800 and 700 iterations. The smooth exponential convergence confirms the stability and efficiency of both methods, with PSO demonstrating clear advantages in speed and final fitness. These observations align with the numerical results in Table 5.

In summary, the results from Table 5 provide strong quantitative evidence for the superiority of PSO over DE in the context of entropy-based multilevel thresholding, as well as the computational advantages of the QR formulation over CR.

We are committed to ensuring the reproducibility of our results. The PSO-QR segmentation algorithm is described in detail within the manuscript, including the quantum encoding process and optimization steps. All evaluation metrics and experimental conditions are clearly specified. While the full implementation and datasets are not publicly hosted at this time, we confirm that these resources will be made available upon reasonable request following publication of this work.

## 5 Conclusion

This paper presents an innovative image segmentation approach that integrates two-dimensional quantum image representation with metaheuristic optimization algorithms, such as Differential Evolution (DE) and Particle Swarm Optimization (PSO). The primary contribution of this work is the novel application of quantum image representation to the 2D histogram, enhancing image thresholding. A comprehensive comparative analysis between classical methods and quantum approaches demonstrates the superior performance of our method.

Empirical testing on twenty benchmark images, evaluated using nine metrics and the Wilcoxon test for statistical significance, establishes the PSO-QR method’s effectiveness, particularly in segmentation accuracy and optimization efficiency. The results highlight the advantages of combining quantum image representation with PSO for precise and computationally efficient thresholding, especially for complex, multimodal problems.

Despite the promising results obtained in simulation, several limitations must be acknowledged regarding the practical implementation of the proposed quantum-based segmentation framework. First, the current approach relies on classical simulation of quantum representations, which inherently limits the scalability and computational speed when processing large-scale image datasets. While the quantum encoding is conceptually efficient, simulating entangled quantum states and density matrices on classical hardware becomes increasingly demanding as the image resolution and the number of gray levels grow. Moreover, the execution of such methods on real quantum hardware remains challenging due to current technological constraints, including limited qubit counts, decoherence, and gate fidelity issues. These hardware limitations hinder the immediate deployment of the method in real-world scenarios, particularly for high-resolution or time-sensitive applications. Additionally, the adaptability of the approach to streaming data or volumetric imaging modalities (e.g., 3D medical scans) is yet to be explored. Future work will focus on optimizing the framework for parallel and hardware-accelerated environments, exploring hybrid quantum-classical solutions, and assessing performance on near-term quantum devices as they become more stable and accessible.

In conclusion, this study advances quantum image representation in image segmentation and optimization, offering promising avenues for future development in both theoretical and practical applications.
